# Supplementary material for: Seasonal Movement Patterns of Urban Domestic Cats Living on the Edge in an African City
Source: Animals (Basel). 2023 Mar 10;13(6):1013. doi: 10.3390/ani13061013 (PMC10044403; doi:10.3390/ani13061013)
Supplement: Supplementary file 1 [file animals-13-01013-s001.zip › animals-2198588-supplementary.pdf]

animals

MDPI

Supplementary Table S1 . Summary statistics on the summer sample of cats GPS-tracked in Cape Town in 2009/2010. Urban (U) or urban-edge (UE) are distinguished, based on their distance from the nearest natural area (U = > 150 m, UE = < 150 m).

Cat Name	Cape Town Suburb	Urban Position	Age (years)	Sex	Hours tracked	MCP area (ha)	95% Kernel area (ha)	50% Kernal area (ha)	Mean distance travelled/day (km)	Max displacement from home (m)
Mountain	Glencairn Heights	UE	5	M	168	31.45	2.71	0.42	13.0	567
Truffles	Glencairn Heights	UE	5	F	168	28.41	3.70	0.58	16.3	417
Drooler	Welcome Glen	UE	6	F	240	47.4	1.47	0.04	10.2	849
Bengie	Hout Bay	UE	12	M	168	51.15	9.09	0.79	16.6	841
Stompie	Noordhoek	UE	9	M	168	33.8	2.76	0.15	16.9	462
Lulu-Belle	Simon's Town	UE	1.25	F	168	36.11	4.22	0.65	16.1	582
Avatar	Noordhoek	UE	12	F	168	24.39	2.27	0.19	12.5	399
Sebastian	Rosebank	U	7	M	168	14.55	1.24	0.09	5.6	413
Mysti	Edgemead	U	5	M	120	15.93	2.46	0.06	4.6	424
Smudge	Fish Hoek	U	11	F	120	0.57	0.14	0.03	1.0	73
Jessica	Claremont	U	10	F	168	49.95	1.00	0.08	4.1	726
Peanut	Lakeside	U	12	M	240	26.94	1.90	0.11	12.6	442.8
Scamp	Vredehoek	U	1.2	M	168	42.75	2.50	0.25	18.1	612.0
Benjy	Claremont	U	7	M	240	39.69	6.55	0.32	17.7	576.8
**Means**							3.00 ± 2.35			

Supplementary Table S2. Summary statistics on the winter sample of cats GPS-tracked in Cape Town in 2011. Urban (U, > 150m) or urban-edge (UE <150 m) are distinguished based on their distance from the nearest natural area.

Cat Name	Cape Town Suburb	Urban Position	Age	Sex	Hours tracked	MCP area (ha)	95% kernel area (ha)	50% kernel area (ha)	Mean distance travelled per day (km)	Max displacement from home (m)
Bella	Welcome Glen	U	5	F	114	2.70	0.78	0.07	4.82	153
Guriume	Welcome Glen	U	15	M	116	2.17	0.58	0.07	5.29	127
Laylah	Glencairn Heights	UE	5.7	F	115	6.01	1.03	0.11	6.60	298
Leekah	Glencairn Heights	UE	8	F	154	4.59	0.56	0.05	9.7	273
Leelu	Glencairn Heights	UE	5.5	F	118	1.94	0.5	0.05	8.57	143
Tankwa	Welcome Glen	UE	3.5	M	138	5.58	1.35	0.13	7.92	193
Tommy	Welcome Glen	U	3	M	147	4.95	1.5	0.11	9.92	168
Wooki	Glencairn Heights	U	12	M	59	2.51	1.01	0.12	2.52	225
Yoda	Welcome Glen	U	6	F	22	0.48	0.48	0.08	1.05	73
**Means**							0.87 ± 0.38			
